# Hydrogen Sulfide Exerted a Pro-Angiogenic Role by Promoting the Phosphorylation of VEGFR2 at Tyr797 and Ser799 Sites in Hypoxia–Reoxygenation Injury

**DOI:** 10.3390/ijms25084340

**Published:** 2024-04-14

**Authors:** Sen Zhang, Yongfeng Cheng, Yining Guan, Jiyue Wen, Zhiwu Chen

**Affiliations:** 1 Department of Pharmacology, School of Basic Medical Sciences, Anhui Medical University, Hefei 230032, China; zhangsen@ahmu.edu.cn (S.Z.); 2145010101@stu.ahmu.edu.cn (Y.G.); 2 Clinical Medical College, Anhui Medical University, Hefei 230012, China; yfcheng@ustc.edu.cn

**Keywords:** angiogenesis, hydrogen sulfide, H/R injury, VEGFR2, Tyr797, Ser799, LC-PRM/MS

## Abstract

The protective effects of hydrogen sulfide (H_2_S) against ischemic brain injury and its role in promoting angiogenesis have been established. However, the specific mechanism underlying these effects remains unclear. This study is designed to investigate the regulatory impact and mechanism of H_2_S on VEGFR2 phosphorylation. Following expression and purification, the recombinant His-VEGFR2 protein was subjected to LC-PRM/MS analysis to identify the phosphorylation sites of VEGFR2 upon NaHS treatment. Adenovirus infection was used to transfect primary rat brain artery endothelial cells (BAECs) with the Ad-VEGFR2^WT^, Ad-VEGFR2Y^797F^, and Ad-VEGFR2^S799A^ plasmids. The expression of VEGFR2 and recombinant Flag-VEGFR2, along with Akt phosphorylation, cell proliferation, and LDH levels, was assessed. The migratory capacity and tube-forming potential of BAECs were assessed using wound healing, transwell, and tube formation assays. NaHS notably enhanced the phosphorylation of VEGFR2 at Tyr797 and Ser799 sites. These phosphorylation sites were identified as crucial for mediating the protective effects of NaHS against hypoxia–reoxygenation (H/R) injury. NaHS significantly enhanced the Akt phosphorylation, migratory capacity, and tube formation of BAECs and upregulated the expression of VEGFR2 and recombinant proteins. These findings suggest that Tyr797 and Ser799 sites of VEGFR2 serve as crucial mediators of H_2_S-induced pro-angiogenic effects and protection against H/R injury.

## 1. Introduction

Stroke, the predominant severe cerebrovascular disease, is the second leading cause of death worldwide, with high rates of morbidity and disability [1]. Ischemic and hemorrhagic stroke are the two main classifications, and cerebral ischemia accounts for approximately 87% of cases [2]. The primary lesion in ischemic stroke (ISs) is cerebral infarction, which is characterized by initial reversible tissue dysfunction and subsequent infarction resulting in the loss of neurons and supportive structures due to inadequate cerebral blood supply [3]. Currently, the management of acute focal stroke involves three main approaches: neuroprotective measures, endovascular thrombectomy, and thrombolytic therapy [4,5]. While neuroprotection is a common strategy for treating both ischemic and hemorrhagic stroke, its clinical effectiveness is limited despite positive results in animal experimental models [6,7]. Hence, it is crucial to have a thorough comprehension of the pathological mechanisms of stroke, and it is imperative to develop enhanced drugs or methods for its treatment.

The concept of the neurovascular unit (NVU), formalized in 2001, emphasizes the intimate relationship between the brain and its vessels [8]. Close communication between neurons and blood vessels provides a new direction for the treatment of IS [9]. The management of IS involves stimulating angiogenesis in the peri-infarct area, which may lead to a substantial reduction in infarct volume, improved neuronal survival, and the restoration of neurovascular network function [10,11]. Following ischemia, angiogenesis is initiated to modify the cerebral microvasculature and collateral circulation, ultimately restoring diminished cerebral perfusion and facilitating the recovery of neurological function [12]. Studies have shown that cerebral ischemia could induce transient angiogenesis [13]. The angiogenesis triggered by cerebral ischemia is frequently dysregulated, leading to uncontrolled vessel formation with unstable permeability and disorganized hierarchy. These abnormal blood vessels, which are prone to leakage and hemorrhage, can exacerbate secondary brain injury and impede the recovery process [14]. Understanding the mechanisms driving dysregulated angiogenesis and promoting vascular stabilization could potentially offer a strategy for improving tissue repair and recovery following cerebral ischemia.

Hydrogen sulfide (H_2_S) is the newest member of the gasotransmitter family, following carbon monoxide (CO) and nitric oxide (NO) [15]. Initially considered an environmental pollutant and toxin, H_2_S is now recognized as an endogenous biological mediator with crucial roles in maintaining homeostasis, regulating physiology, and impacting disease [16,17]. Endogenous H_2_S is produced enzymatically by three enzymes: cystathionine-β-synthase (CBS), cystathionine-γ-lyase (CSE), and 3-mercaptopyruvate sulfurtransferase (3-MST) [18]. Endogenously produced H_2_S and various H_2_S donors have been implicated in the promotion of angiogenesis both in vivo and in vitro studies, indicating potential translational opportunities [18,19,20]. Despite the compelling evidence of H_2_S involvement in angiogenesis, the specific mechanisms and receptors that regulate the biology of H_2_S in this context remain elusive.

Vascular endothelial growth factors (VEGFs) and their receptors (VEGFRs) are the foremost specific factors in vasculogenesis and angiogenesis at present, playing important roles in both physiological and pathological processes [21]. VEGFR2, the principal receptor for VEGF, is predominantly found in vascular endothelial cells and plays a crucial role in angiogenesis [22,23]. VEGF-activated VEGFR2 facilitates and mediates distinct downstream signaling pathways, which are associated with various cellular responses, including proliferation, migration, survival, and permeability [23,24,25]. Previous research has demonstrated direct and indirect interactions between H_2_S and VEGFR2. VEGFR2 may serve as a receptor for H_2_S and could be directly activated by H_2_S [26]. H_2_S can also regulate the expression of VEGF, activate the protein kinase Akt, and affect the production of other signaling molecules, such as nitric oxide (NO) and endothelial nitric oxide synthase (eNOS), which can further modulate VEGFR2 signaling via an indirect manner [27]. The pro-angiogenic effect of H_2_S might be associated with the activation of the VEGF/VEGFR2 signaling pathway. However, the exact mechanism remains to be elucidated. Liquid chromatography–parallel reaction monitoring/mass spectrometry (LC-PRM/MS) is a new generation of mass spectrometry technology that has important applications and potential in biomedical research and protein research. With the characteristics of high throughput, high selectivity, and high sensitivity, LC-PRM/MS technology can accurately measure the abundance and change of proteins, providing a powerful tool for proteome analysis [28]. This study is designed to explore the regulatory impact of H_2_S on the phosphorylation of VEGFR2 at other potential sites when using the LC-PRM/MS method and its underlying regulation in angiogenesis following IS.

## 2. Results

### 2.1. Expression and Purification of His-VEGFR2 Recombinant Protein

**Figure 1:**
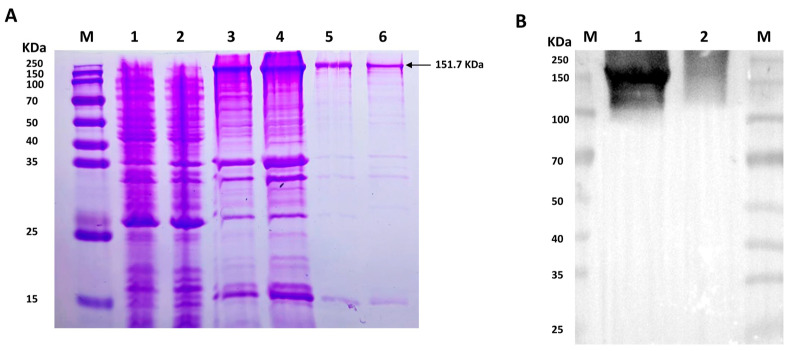
The figure shows the expression and purification of His-VEGFR2 in *E. coli* and Western blot determination. (**A**) Coomassie brilliant blue staining for the expression of recombinant His-VEGFR2 protein. M: Marker; 1: the lysate of non-transfected *E. coli*; 2: the lysate of uninduced *E. coli* transfected with His-VEGFR2-pET28a (+); 3: the total lysate of induced *E. coli*; 4: the precipitate of lysate from the induced *E. coli*; 5: the purified inclusion body protein; 6: the final purified protein. (**B**) Western blot detection for purified His-VEGFR2 protein. M: Marker; 1: purified His-VEGFR2 protein; 2: uninduced lysate of *E. coli* for control.

To detect the potential phosphorylation site of VEGFR2 upon H_2_S treatment, prokaryotic plasmid His-VEGFR2-pET28a (+) was constructed and transformed to *E. coli* to express His-tagged VEGFR2 protein. The SDS-PAGE analysis shown in Figure 1A revealed a distinct band in both the total lysate and precipitate of the IPTG-induced *E. coli*, with a molecular weight of approximately 151.7 KDa, which is consistent with the expected molecular weight of the target protein. In contrast, no specific band was observed in non-transfected *E. coli* and uninduced but transfected *E. coli*. After purification, the results confirmed the fusion protein’s expected size, indicating high purity for subsequent purification processes. The recombinant protein was successfully produced through renaturation and Ni-NTA purification. Western blot analysis in Figure 1B confirmed the successful acquisition of the purified recombinant protein.

### 2.2. Effect of NaHS on the Related Sites Phosphorylation of His-VEGFR2 In Vitro

**Figure 2:**
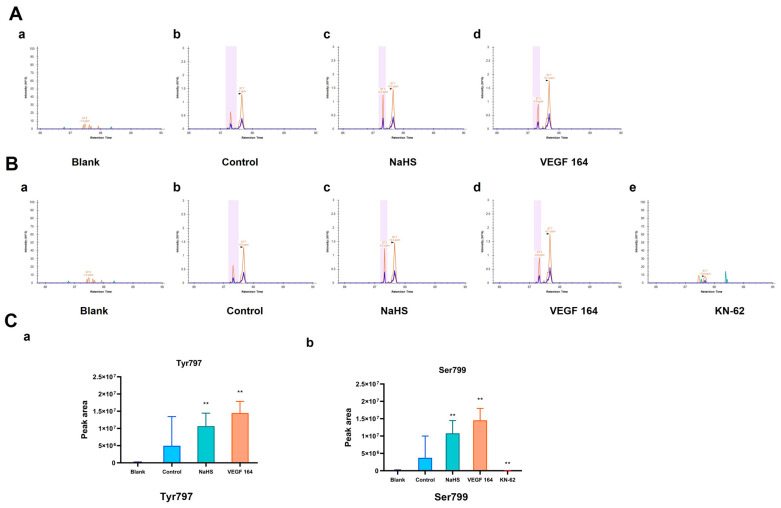
The figure shows the effects of NaHS on the phosphorylation of His-VEGFR2 at Tyr797 and Ser799 through LC-PRM/MS in vitro. The comparison of phosphorylation levels between each group was based on the total intensity of each peptide fragment, and the retention time was marked in the spectrograms. (**A**) Phosphorylation at Tyr797. The results indicated that VEGFR2 795–812: TGYLSIVMDPDELPLDER in the blank group (**a**), TG (pho) YLSIVMDPDELPLDER in the control group (**b**), NaHS group (**c**) and VEGF 164 group (**d**). (**B**) Phosphorylation at Ser799. The results indicated that VEGFR2 795–812: TGYLSIVMDPDELPLDER in the blank group (**a**) and KN-62 group (**e**); TGYL (pho) SIVMDPDELPLDER in the control group (**b**), NaHS group (**c**) and VEGF 164 group (**d**). (**C**) The quantitative results of phosphorylation for Tyr797 (**a**) and Ser799 (**b**). Data represent mean ± SD. Compared to the control group, ** *p* < 0.01 on one-way ANOVA. *n* = 3.

To detect the potential phosphorylation sites of His-VEGFR2 recombinant protein, the LC-PRM/MS assay, a higher-resolution and accurate technique, was used to quantitate the phosphorylation level of VEGFR2 under the H_2_S donor NaHS treatment. To accomplish this goal, we first used Skyline 22.2.0, a freely available software tool (online at http://skyline.maccosslab.org, accessed on 10 February 2023). As depicted in Figure 2, no phosphorylation was detected at any site in the blank groups containing only recombinant protein without ATP. In the control group where ATP was present, phosphorylation occurred at Tyr797, indicating the autophosphorylation of VEGFR2 in this case. Meanwhile, NaHS and VEGF 164 significantly increased the phosphorylation of His-VEGFR2 at Tyr 797, suggesting that NaHS functions as a VEGFR2 activator, similar to VEGF 164 (Figure 2A). In addition, phosphorylation at serine site Ser799 was also observed in the control group. Considering that VEGFR2 is a typical receptor tyrosine kinase, we hypothesized that it may be related to the purity of the recombinant protein. The identification of protein components using the shotgun method indicated the presence of the typical serine/threonine kinase CaMK IV (presented in the Supplementary Materials). Therefore, we used KN-62, a specific inhibitor of CaMK IV, for comparison. Compared to the control group, phosphorylation at Ser799 was no longer found in the KN-62 group, while NaHS and VEGF 164 induction significantly enhanced the phosphorylation of VEGFR2 at the Ser799 site (Figure 2B). The discovery of Ser799 signifies the identification of a previously unknown phosphorylation site. In combination, NaHS has been shown to enhance the phosphorylation of VEGFR2 at both Tyr797 and Ser799.

### 2.3. Transfection of Recombinant Eukaryotic Plasmid Ad-VEGFR2^WT^, Ad-VEGFR2^Y797F^ and Ad-VEGFR2^S799A^ by Adenovirus in Primary Rate Brain Endothelial Cells (BAECs)

**Figure 3:**
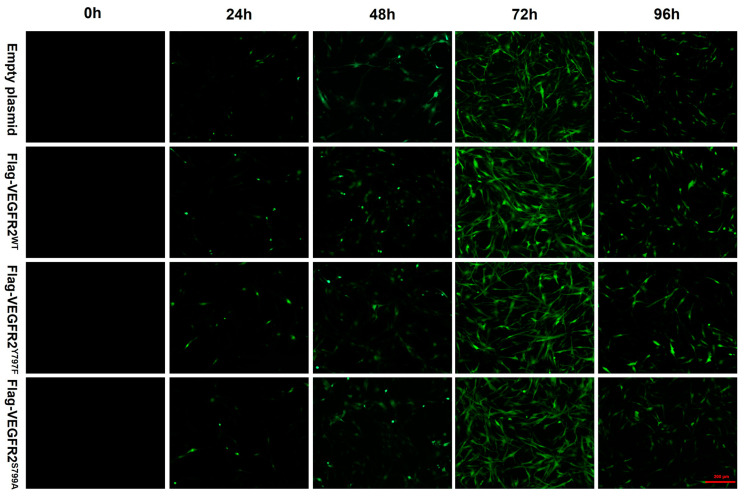
The figure shows the representative image of BAECs transfected with empty plasmid, Ad-VEGFR2^WT^, Ad-VEGFR2^Y797F^, and Ad-VEGFR2^S799A^ plasmids. The transfection efficiency of the plasmids was determined by the expression of green fluorescent proteins (GFPs). GFPs showed green fluorescence. (×100; Scale bar: 200 μm).

To investigate the effect of NaHS on the function of BAECs through the modulation of VEGFR2 phosphorylation at Tyr797 and Ser799, wild-type plasmids Ad-VEGFR2^WT^ and point mutant plasmids Ad-VEGFR2^Y797F^ and Ad-VEGFR2^S799A^ were constructed by Sangon Biotech (Shanghai) Co. (Shanghai, China) The tyrosine (Y) residue at the 797 site was mutated into phenylamine (F) residue and the serine (S) residue at the 799 site was mutated into alanine (A) residue. Three types of plasmids and an empty plasmid were transfected into BAECs through adenovirus infection. Transfection efficiency was assessed by the intensity of green fluorescent protein (GFP). As depicted in Figure 3, GFP expression was observed in BAECs post-transfection, with significantly higher fluorescence intensity at 72 h compared to other time points, indicating maximal transfection efficiency at 72 h. Consequently, BAECs transfected for 72 h were selected for subsequent experiments.

### 2.4. NaHS Promoted the Expression of Flag-VEGFR2 and VEGFR2 in Transfected BAECs

**Figure 4:**
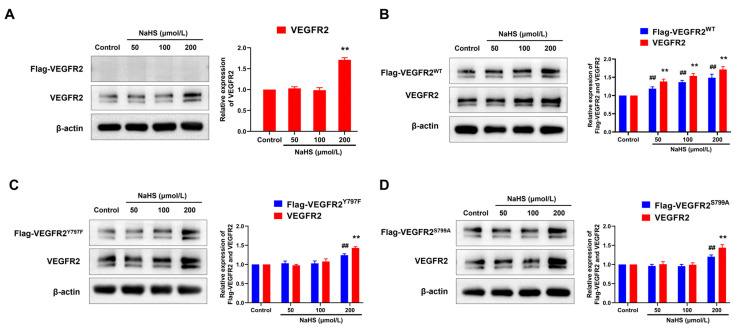
The figure shows the effects of NaHS on the expression of VEGFR2, Flag-VEGFR2^WT^, Flag-VEGFR2^Y797F^, and Flag-VEGFR2^S799A^ in the transfected BAECs. (**A**) Empty plasmid-transfected BAECs. ** *p* < 0.01 vs. control group. (**B**) Ad-VEGFR2^WT^ transfected BAECs. ^##^ *p* < 0.01 vs. control group (Flag-VEGFR2^WT^, anti-Flag antibody). ** *p* < 0.01 vs. control group (total VEGFR2, anti-VEGFR2 antibody). (**C**) Ad-VEGFR2^Y797F^ transfected BAECs. ^##^ *p* < 0.01 vs. control group (Flag-VEGFR2^Y797F^, anti-Flag antibody). ** *p* < 0.01 vs. control group (total VEGFR2, anti-VEGFR2 antibody). (**D**) Ad-VEGFR2^S799A^ transfected BAECs. ^##^ *p* < 0.01 vs. control group (Flag-VEGFR2^S799A^, anti-Flag antibody). ** *p* < 0.01 vs., control group (total VEGFR2, anti-VEGFR2 antibody). The data were fold-changed to the control group. Data represent mean ± SD. One-way ANOVA. *n* = 3.

To elucidate the regulatory mechanism of H_2_S in angiogenesis, we assessed the expression levels of VEGFR2 and recombinant protein Flag-VEGFR2, which is fused with a Flag protein at the C-terminal region. As shown in Figure 4, only VEGFR2 and no Flag-VEGFR2 protein were detected in empty plasmid-transfected BAECs. Treatment with NaHS at concentrations of 200 μmol/L increased VEGFR2 expression (Figure 4A). In BAECs transfected with a recombinant eukaryotic plasmid, both VEGFR2 and Flag-tagged VEGFR2 (including Flag-VEGFR2^WT^, Flag-VEGFR2^Y797F^ and Flag-VEGFR2^S799A^) were observed, conforming successful transfection. Treatment with NaHS at concentrations of 50, 100, and 200 μmol/L remarkably increased the expression of VEGFR2 and Flag-VEGFR2^WT^ in Ad-VEGFR2^WT^ transfected BAECs (Figure 4B). However, the expression of VEGFR2, Flag-VEGFR2^Y797F^, and Flag-VEGFR2^S799A^ in Ad-VEGFR2^Y797F^ and Ad-VEGFR2^S799A^ transfected BAECs was only elevated at the concentration of 200 μmol/L (Figure 4C,D). These results indicate that H_2_S promoted the expression of VEGFR2 and the recombinant proteins Flag-VEGFR2^WT^, Flag-VEGFR2^Y797F^, and Flag-VEGFR2^S799A^ in BAECs, which might represent a potential mechanism underlying the pro-angiogenic effect of H_2_S.

### 2.5. The Mutation at Tyr797 and Ser799 of VEGFR2 Attenuated the NaHS-Induced Activation of Akt Signaling in BAECs

**Figure 5:**
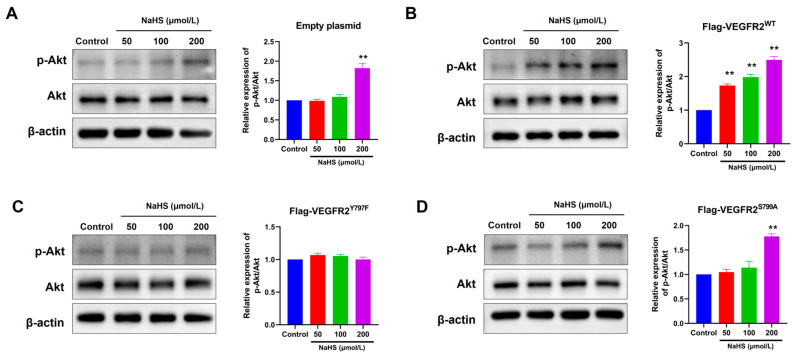
Tyr797 and Ser799 mediated the promotion of NaHS on the phosphorylation of Akt in transfected BAECs. The phosphorylated Akt was detected using an anti-pSer473-Akt antibody. (**A**) Empty plasmid-transfected BAECs. ** *p* < 0.01 vs. control group. (**B**) Ad-VEGFR2^WT^-transfected BAECs. ** *p* < 0.01 vs. control group. (**C**) Ad-VEGFR2^Y797F^-transfected BAECs. (**D**) Ad-VEGFR2^S799A^-transfected BAECs. ** *p* < 0.01 vs. control group. The data were fold-changed to the control group. The data represent mean ± SD. One-way ANOVA. *n* = 3.

To confirm the influence of H_2_S on VEGFR2 downstream signaling in BAECs transfected with different plasmids, we observed the phosphorylation of Akt at Ser473, a recognized downstream pathway of VEGFR2 involved in angiogenesis. In Figure 5, NaHS stimulation induced moderate Akt (p-Ser473) phosphorylation in BAECs transfected with an empty plasmid (Figure 5A). This phosphorylation notably increased in BAECs overexpressing Flag-VEGFR2^WT^ (Figure 5B). Conversely, NaHS-stimulated Akt phosphorylation levels in BAECs overexpressing the mutant VEGFR2^Y797F^ and VEGFR2^S799A^ resembled those in BAECs expressing only endogenous VEGFR2, yet they were significantly lower than in BAECs overexpressing Flag-VEGFR2^WT^ (Figure 5C,D). The findings indicate that the phosphorylation of Akt induced by NaHS is mediated by Tyr797 and Ser799 of VEGFR2. This is supported by the observation that the overexpression of mutant proteins at these sites does not lead to higher levels of Akt activation compared to BAECs transfected with an empty plasmid.

### 2.6. Tyr797 and Ser799 Are Involved in the Protective Effect of NaHS against Hypoxia–Reoxygenation (H/R) Injury in BAECs

Since the Tyr797 and Ser799 of VEGFR2 mediated the promotion of NaHS in activating Akt signaling, and it has also been reported that Akt signaling promotes the proliferation of endothelial cells, the protective role of NaHS against H/R injury in BAECs was investigated in this present study.

**Figure 6:**
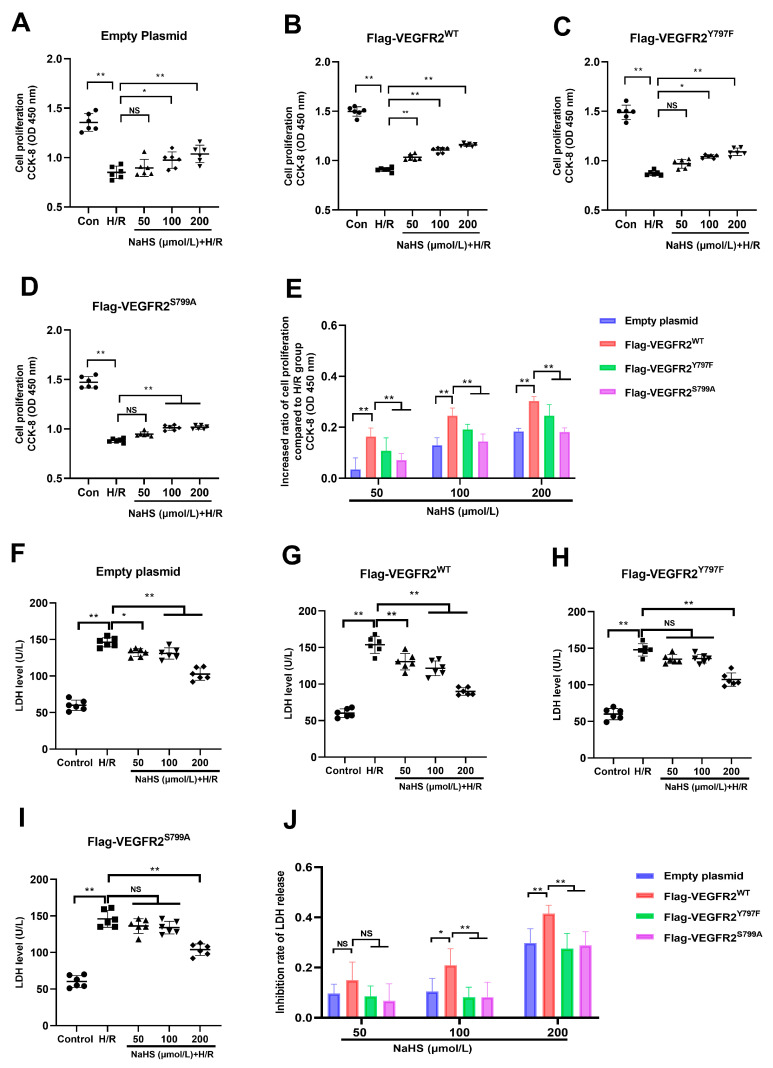
Tyr797 and Ser799 mediated the effects of NaHS against H/R injury of BAECs. (**A**) Cell proliferation of empty plasmid-transfected BAECs. (**B**) Cell proliferation of Ad-VEGFR2^WT^-transfected BAECs. (**C**) Cell proliferation of Ad-VEGFR2^Y797F^-transfected BAECs. (**D**) Ad-VEGFR2^S799A^-transfected BAECs. (**E**) Increased ratio of cell proliferation after NaHS treatment. (**F**) LDH level of empty plasmid-transfected BAECs. (**G**) LDH level of Ad-VEGFR2^WT^-transfected BAECs. (**H**) LDH level of Ad-VEGFR2^Y797F^-transfected BAECs. (**I**) LDH level of Ad-VEGFR2^S799A^-transfected BAECs. (**J**) Inhibition ratio on increased LDH levels. * *p* < 0.05, ** *p* < 0.01 vs. the indicated group. NS: No Significance. Data represent means ± SD. One-way ANOVA, *n* = 6.

As shown in Figure 6, exposure to H/R caused notable injury compared to the control group. The diminished proliferation capacity and raised LDH levels in the supernatant of BAECs were evident in all H/R groups, pointing to this observation. NaHS prevented the decline in cell proliferation at concentrations of 100 and 200 μmol/L and suppressed the elevation of LDH levels at concentrations of 50, 100, and 200 μmol/L in BAECs transfected with an empty plasmid (Figure 6A,F). In BAECs transfected with Ad-VEGFR2^WT^, treatment with 50, 100, and 200 μmol/L of NaHS prevented the reduction in proliferation capacity and elevation of LDH levels, as shown in Figure 6B,G. In BAECs transfected with Ad-VEGFR2^Y797F^ or Ad-VEGFR2^S799A^, NaHS at concentrations of 100 and 200 μmol/L was found to restore proliferation ability, while only 200 μmol/L of NaHS was effective in lowering increased LDH levels (Figure 6C,D,H,I).

In addition to comparing the differences between the NaHS treatment groups and the H/R group, we calculated the inhibitory rate of NaHS on the decreased proliferation ability and increased LDH levels in all plasmid-transfected BAECs and then compared the differences between them. In BAECs transfected with Ad-VEGFR2^WT^, NaHS at concentrations of 100 and 200 μmol/L showed a higher inhibitory ratio on reduced cell proliferation compared to BAECs transfected with Ad-VEGFR2^Y797F^ or Ad-VEGFR2^S799A^, as illustrated in Figure 6E. Moreover, BAECs transfected with Ad-VEGFR2^Y797F^ or Ad-VEGFR2^S799A^ exhibited a reduced inhibitory ratio in terms of NaHS on elevated LDH levels at 100 and 200 μmol/L when contrasted with cells transfected with Ad-VEGFR2^WT^ (Figure 6J).

These findings suggest the involvement of Tyr797 and Ser799 of VEGFR2 in protecting NaHS against H/R injury in BAECs.

### 2.7. Tyr797 and Ser799 Mediated the Promoting Effect of NaHS on BAECs Migration

**Figure 7:**
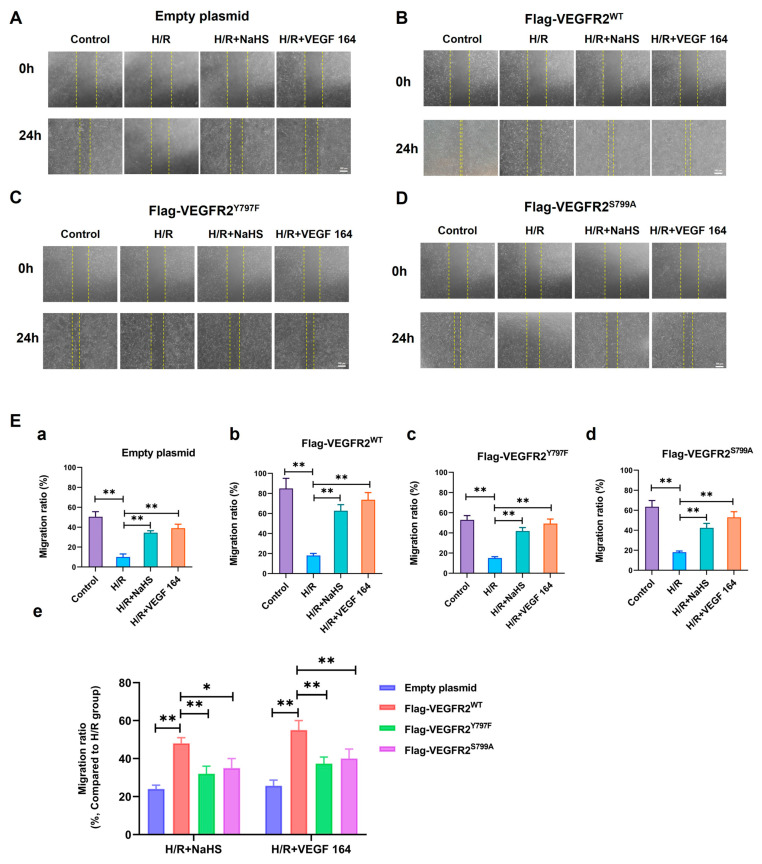
Tyr797 and Ser799 mediated the promoting effect of NaHS on BAECs migration in wound healing assay. The migration ability was first measured via wound healing assay. (**A**–**D**) represent empty plasmid, Ad-VEGFR2^WT^, Ad-VEGFR2^Y797F^, and Ad-VEGFR2^S799A^ transfect BAECs, respectively (×50; Scale bar: 100 μm). The yellow lines indicate boundary of the scratch at 24 h. (**E**) (**a**–**d**): The comparison of migration ratio after transfection with different plasmids, ** *p* < 0.01 compared with indicated group. (**e**): The comparison of difference values in migration ratio between NaHS and VEGF 164 groups minus H/R group. * *p* < 0.05, ** *p* < 0.01 compared with the indicated group. Data were presented as means ± SD. One-way ANOVA, *n* = 3.

**Figure 8:**
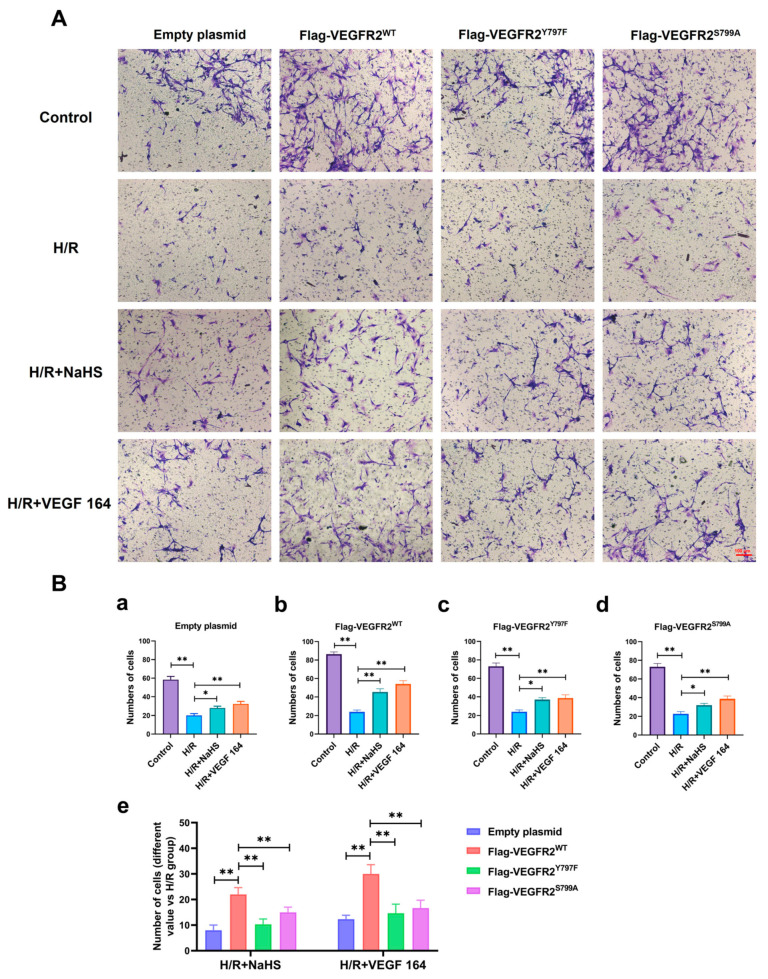
Tyr797 and Ser799 mediated the promoting effect of NaHS on BAECs migration in a transwell assay. (**A**) Representative images of BAECs passing through the chamber after transfection with different plasmids (×100; Scale bar: 100 μm). (**B**) (**a**–**d**): The comparison of cell numbers after transfection with different plasmids, * *p* < 0.05, ** *p* < 0.01 compared with the indicated group. (**e**): A comparison of different values in cell terms of number between NaHS and VEGF 164 groups minus the H/R group. ** *p* < 0.01, compared with indicated group. All data represent means ± SD, one-way ANOVA, *n* = 3.

The phosphorylation of VEGFR2 at Tyr797 and Ser799 sites induced by H_2_S plays a promoting role in the proliferation of BAECs. Consequently, we investigated the involvement of Tyr797 and Ser799 in regulating the migratory capacity of BAECs following NaHS treatment. In wound healing and transwell assays, the findings indicated that the migratory capacity of BAECs transfected with an empty plasmid was partially restored by NaHS or VEGF 164 treatment compared to the H/R group, which had already been diminished under hypoxic conditions. Furthermore, the recuperation of migratory ability in BAECs transfected with Ad-VEGFR2^WT^ was significantly enhanced under NaHS or VEGF 164 treatment compared to the H/R group. Additionally, in BAECs transfected with Ad-VEGFR2^Y797F^ or Ad-VEGFR2^S799A^, the migratory ability was also partially recovered compared to the H/R group (Figure 7A–D,E(a–d) and Figure 8A,B(a–d)).

To compare the recuperative migratory capacity among BAECs transfected with different plasmids, we collected the respective values of the recovered migratory rate. The migratory rate of BAECs transfected with Ad-VEGFR2^Y797F^ or Ad-VEGFR2^S797A^ was significantly weaker compared to BAECs transfected with Ad-VEGFR2^WT^ under both NaHS and VEGF 164 treatment, but higher than that in BAECs transfected with an empty plasmid (Figure 7E(e) and Figure 8B(e)). These findings suggest that Tyr797 and Ser799 of VEGFR2 play a role in mediating the promoting effect of NaHS on BAEC migration.

### 2.8. Tyr797 and Ser799 Mediated the Tube Formation of BAECs Induced by NaHS In Vitro

**Figure 9:**
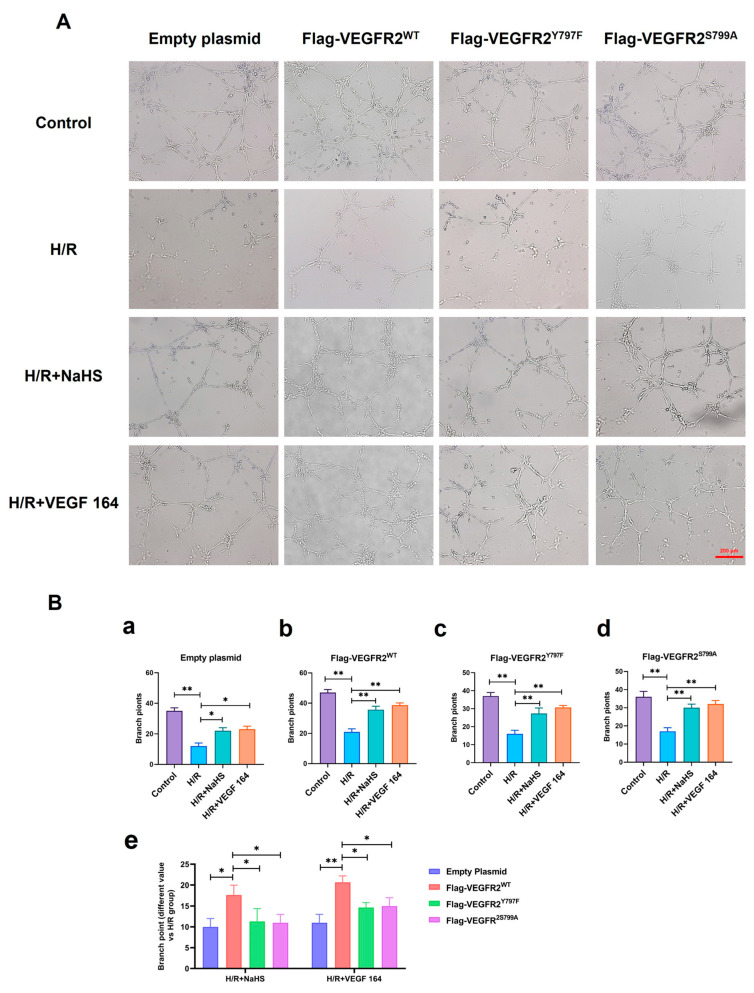
Tyr797 and Ser799 mediated the tube formation of BAECs induced by NaHS in vitro. (**A**) Representative capillary-like structures in BAECs after transfection with different plasmids (×100; Scale bar: 200 μm). (**B**) (**a**–**d**): The comparison of difference value in branch point numbers after transfection with different plasmids, * *p* < 0.05, ** *p* < 0.01 compared with H/R group. (**e**): The comparison of difference value in branch points when NaHS and VEGF 164 group minus H/R group. * *p* < 0.05, ** *p* < 0.01 compared with Ad-VEGFR2^WT^ group. All data represent means ± SD, one-way ANOVA, *n* = 3.

The roles of Tyr797 and Ser799 in H_2_S-induced angiogenesis of BAECs were previously unclear. To address this, we conducted a Matrigel tube formation assay. All H/R groups in BAECs transfected with empty plasmid, Ad-VEGFR2^WT^, Ad-VEGFR2^Y797F^, and Ad-VEGFR2^S799A^ plasmids, formed fewer tube-like networks compared to their corresponding control groups. Treatment with NaHS or VEGF 164 significantly improved the number of branch points (tube formation ability) compared to the H/R group (Figure 9A). To compare the differences between wild-type and mutant plasmids, we calculated the difference value in terms of tube branch point number between the treated groups and the H/R groups. The results indicated that BAECs transfected with Ad-VEGFR2^WT^ displayed enhanced tube formation ability (the improved number of branch points) compared to those transfected with Ad-VEGFR2^Y797F^ or Ad-VEGFR2^S799A^ when exposed to NaHS or VEGF 164 (Figure 9B). These findings demonstrate that Tyr797 and Ser799 of VEGFR2 mediate the angiogenic role of BAECs induced by H_2_S.

## 3. Discussion

The pro-angiogenic effect of VEGF on endothelial cells (ECs) relies on the VEGFR2 dimerization and subsequent activation of a downstream signaling pathway [29]. Despite the well-established roles of VEGFR2 in angiogenesis, the specific regulatory mechanism and action site of H_2_S are still unknown. In this study, we demonstrated that H_2_S directly activates VEGFR2 by inducing phosphorylation at Tyr797 and Ser799 sites in vitro. This activation is pivotal in mediating the protective effects of H_2_S against H/R injury, as demonstrated by the inhibition of decreased cell proliferation and increased LDH levels of BAECs. Moreover, H_2_S enhances the migratory and tube formation abilities of BAECs, which are impaired by the mutations in Tyr797 and Ser799 sites of VEGFR2. These results demonstrated the role of Tyr797 and Ser799 sites of VEGFR2 in angiogenesis and provide a theoretical basis for clarifying the mechanism of H_2_S in promoting angiogenesis.

Angiogenesis, which refers to the growth of blood vessels from the pre-existing vasculature, is a crucial process for embryonic and postnatal development, tissue repair, and reproductive functions [30]. It is also implicated in major disease processes, with the typical one being ischemic stroke [31,32]. Angiogenesis improves cerebral blood circulation and restores cerebral blood flow after ischemia, thereby supplying power for extensive neural activities in the brain [33]. The VEGF family and their receptor VEGFR1-R3 are extensively studied pathways in vasculature development, playing crucial roles in both developmental and pathological angiogenesis [34]. VEGFR2 is a major mediator of the physiological and pathological effects of VEGFs, which mediates EC proliferation and migration, sprouting angiogenesis through different downstream signaling [35,36].

Post-translational modifications (PTMs) are covalent processes that change the properties of a protein through proteolytic cleavage and the addition of modifying groups to one or more amino acids [37,38]. Phosphorylation, a widely studied protein modification, involves the enzymatic addition of a phosphate group to serine, threonine, and tyrosine residues. It is a reversible process, which is typically used to switch various biological processes on and off, such as the cell cycle, metabolism, and regulation of receptors [39]. The presence of multiple potential phosphorylation sites in VEGFR2 suggests a complex interaction, yet the specific interactions between H_2_S and VEGFR2 remain poorly elucidated. In this study, a prokaryotic plasmid His-VEGFR2-pET-28a (+) was constructed to express wild-type His-VEGFR2 protein in *E. coli*. The aim was to detect the modulation of H_2_S on VEGFR2 and explore potential phosphorylation sites. The Coomassie brilliant blue staining and Western blot assays revealed the presence of recombinant His-VEGFR2 protein in the lysate precipitation of *E. coli*. This suggests that His-VEGFR2 protein exists within an inclusion body. The inclusion bodies were renatured and purified using Ni-NTA purification, resulting in the successful acquisition of recombinant protein.

LC-PRM/MS technology plays a crucial role in current proteomic research due to its superior resolution and mass accuracy. It allows for the simultaneous monitoring of all transitions as a full MS/MS scanning profile, thus providing enhanced selectivity and confidence in the quantitation of each analyzed target protein [40,41,42]. We observed that in the absence of ATP, no phosphorylation was detected, while the presence of ATP induced phosphorylation in Tyr797, indicating that the phosphorylation of Tyr797 happened in an autophosphorylation manner, reflecting its characteristic as an RTK. Furthermore, our study showed that the treatment of exogenous H_2_S donor, NaHS and VEGF 164, a ligand for VEGFR2, significantly promoted phosphorylation at Tyr797. Interestingly, phosphorylation at Ser799 in the absence of serine kinase reminded us to identify the recombinant protein component via the shotgun method. The result suggests that CaMK IV, one kind of serine/threonine kinase, was contained in a purified recombinant protein. KN-62, the specific inhibitor of CaMK IV, could inhibit phosphorylation at Ser799 in our experiment. Meanwhile, the treatment of NaHS and VEGF 164 significantly improved the phosphorylation of VEGFR2 at Ser799. Some previous studies have reported that H_2_S directly activated VEGFR2 and increased the kinase activity of VEGFR2 [26]. These results indicate that H_2_S could promote the phosphorylation of VEGFR2 at Tyr797 and Ser799. Ser799, as a phosphorylation site of VEGFR2, was first reported.

The interaction between H_2_S and VEGFR2 signaling has been extensively studied, demonstrating their collaborative role in promoting angiogenesis. Previous studies have proposed that H_2_S serves as a downstream effector of VEGF signaling [43,44,45], yet it can also function as an upstream regulator of VEGF [46,47,48], depending on the conditions or models studied. Endogenously produced H_2_S by CBS increases the stability and transcriptional activity of specificity protein 1 (Sp1) [49], enhancing VEGFR2 transcription and expression levels. In this study, the recombinant plasmids Ad-VEGFR2^WT^, Ad-VEGFR2^Y797F^ and Ad-VEGFR2^S799A^ were successfully transfected to BAECs, which was confirmed via GFP fluorescence and Flag-tagged VEGFR2 detection. Consistent with previous studies, NaHS promoted the expression of VEGFR2 in each plasmid-transfected BAEC. As for Flag-VEGFR2 expression, NaHS not only enhanced the expression of Flag-VEGFR2^WT^ at concentrations of 50, 100, and 200 μmol/L but also facilitated the expression of Flag-VEGFR2^Y797F^ and Flag-VEGFR2^S799A^ at 200 μmol/L. This means that both Tyr797 and Ser799 did not mediate the promotion of H_2_S on the expression of VEGFR2 in BAECs. Furthermore, NaHS significantly induced the phosphorylation of Akt in the Ad-VEGFR2^WT^ transfected BAECs, and this induction was notably impaired after Ad-VEGFR2^Y797F^ or Ad-VEGFR2^S799A^ transfection. Previous research has demonstrated that VEGFR2 was phosphorylated by H_2_S in a time-dependent manner, corresponding with the downstream phosphorylation of Akt (Ser473). However, it was confirmed that H_2_S directly activated the kinase activity of VEGFR2 and not Akt in a cell-free system [26]. Building upon the previously mentioned results regarding the H_2_S promotion of VEGFR2 phosphorylation at Tyr797 and Ser799, our study elucidated that H_2_S activates VEGFR2 signaling by inducing the phosphorylation of VEGFR2 at Tyr797 and Ser799 as well as downstream Akt phosphorylation.

During angiogenesis, ECs undergo differentiation into tip and stalk cells. Tip cells, characterized by high motility, guide the nascent sprouts, while stalk cells contribute to the structural support of the nascent capillary [21,50]. The formation of tip cells is mechanistically reliant on VEGFR2 phosphorylation. In this study, our results revealed that H_2_S promoted VEGFR2 phosphorylation at the Tyr797 and Ser799 sites and significantly improved the proliferation and migration of BAECs in the Ad-VEGFR2^WT^-transfected group following H/R injury. H_2_S also increased the tube formation ability of Ad-VEGFR2^WT^-transfected BAECs, which was proved by the elevated number of branch points. Notably, this improvement, including proliferation and migratory and tube formation abilities, was impaired by the mutation of VEGFR2 at Tyr797 and Ser799, underscoring the significance of these sites in mediating the observed effects. Thereby, our findings uncovered novel phosphorylation sites, along with their associated functions and mechanisms in angiogenesis.

The study demonstrates that H_2_S promotes VEGFR2 phosphorylation at Tyr797 and Ser799 sites, as well as Akt phosphorylation, a key downstream signaling pathway. The activation of VEGFR2 turns on intracellular signaling pathways that are crucial to endothelial biology. These include the phospholipase Cγ (PLCγ)—extracellular regulated protein kinases (ERKs) 1/2 pathway, which has a central role during vascular development and adult arteriogenesis [51]; the phosphatidylinositol 3 kinase (PI3K)—Akt pathway, which is crucial for cell survival and the regulation of vasomotion and barrier function [52]. H_2_S exhibits protective and pro-angiogenic effects on BAECs following H/R injury through VEGFR2 phosphorylation at Tyr797 and Ser799. It remains to be determined whether H_2_S directly activates VEGFR2 via Tyr797 and Ser799 phosphorylation or if other mechanisms are involved. The juxtmembrane domains (JMDs, 790–833 aa), located adjacent to the lipophilic membrane where VEGFR2 is anchored, have been reported to regulate kinase activity in multiple ways. The JMDs of VEGFR2 contain the first phosphorylation site of Tyr801 (Tyr797 in rats), which is crucial for the autophosphorylation rate of VEGFR2 [53]. The unphosphorylated JMD autoinhibits kinase activity by interacting with the activation loop (A-loop) in kinase domain 2 (TKD2). Therefore, it is hypothesized that the phosphorylation of JMD at Tyr797 and Ser799 may disrupt this interaction with the A-loop, facilitate reorientation of the activation loop, and induce an enzymatically active conformation.

## 4. Materials and Methods

### 4.1. Regents

NaHS (161527) and IPTG (I6758) were obtained from Sigma. Anti-Flag antibody (ab205606) was purchased from Abcam (Cambridge, UK). ATP (#9804), Kinase Buffer (10×) (#9802), anti-VEGFR2 antibody (#9698), and anti-phospho-Akt (Ser473) (#4060) were provided by Cell Signaling Technology (Danvers, MA, USA). Anti-Akt antibody (sc-5298) was purchased from Santa Cruz (Dallas, TX, USA). Anti-β-actin antibody (TA-09) was purchased from ZSGB-BIO (Beijing, China). CCK-8 kit (BS350B) was purchased from Biosharp (Hefei, China). An LDH assay kit (A020-2-2) was provided by Nanjing Jiancheng Bioengineering Institute (Nanjing, China). KN-62 (S7422) was purchased from Selleck (Houston, TX, USA).

### 4.2. Expression and Purification of the Prokaryotic Recombinant Proteins of His-VEGFR2

The prokaryotic His-VEGFR2-pET28A (+) plasmid was constructed by Gene Create Biological Engineering, CO. (Wuhan, China). *E. coli* was provided by the same institution. The sequencing results confirmed the successful construction of the plasmid. Subsequently, the plasmid was transformed into *E. coli*. Briefly, 10 μL of plasmid was added to 100 μL of *E. coli* and kept on ice immediately for 20 min. *E. coli* was placed in water at 42 °C for 90 s. Next, 800 μL of preheated liquid LB medium was added to the *E. coli*, and the mixture was then cultured at 37 °C for 24 h. The bacteria were inoculated into LB solid medium after centrifugation and resuscitation. A positive clone was chosen and then inoculated into LB liquid medium and cultured further at 37 °C. The medium was added with IPTG at a final concentration of 1.0 mM, when the OD value of the bacteria reached 0.6. After 6 h of induction, the bacteria were centrifuged and resuspended by cold NTA-0 buffer solution with lysozyme at a 0.1 mg/mL final concentration. The bacteria were then broken using ultrasound, and the supernatant and precipitate were separated through centrifugation. Sodium dodecyl sulfate–polyacrylamide gel electrophoresis (SDS-PAGE) analysis revealed the presence of the recombinant protein in the precipitate. Following renaturation and Ni column purification, the purified His-VEGFR2 protein was obtained, which was subsequently confirmed using Coomassie blue stain and Western blot.

### 4.3. In Vitro Phosphorylation Assay

The in vitro phosphorylation assay was performedto detect the phosphorylation of VEGFR2. Briefly, about 150 μL of VEGFR2 protein (50 μg), 10 μL of 10 mM ATP (100 μM), with or without 4 μL of 5 μg/mL VEGF 164 (100 ng/mL), 10 μL of 2 mM NaHS (100 μM) or 10 μL of 200 μM KN-62 (10 μM) in the inhibitor group were sequentially added to kinase buffer and mixed fully. The mixture was shaken for 30 min at 37 °C. The reaction was terminated with 30 mM EDTA solution. The solution was then sent to AIMSMASS Co., Ltd. (Shanghai, China) for LC-PRM/MS assay to detect the phosphorylation of VEGFR2 at the related sites.

### 4.4. LC-PRM/MS

Proteolysis was conducted as follows: DTT was added to each sample, reaching a final concentration of 100 mM. The samples were then boiled in water for 15 min and subsequently cooled to room temperature. The mixed sample containing 200 μL of UA buffer (8 M of urea, 150 mM Tris-HCl, pH 8.0) was transferred to a 10 KD ultrafiltration tube and then centrifuged at 14,000× *g* for 30 min. Following this, 200 µL of UA was added, and the mixture was centrifuged at 14,000× *g* for 30 min. After discarding the filtrate from the collection tube, the proteins were alkylated with IAA (50 mM IAA in UA) and then incubated for 30 min in the dark. We discarded the flow-through after centrifuging at 14,000× *g* for 20 min. Subsequently, 100 µL of UA was added, followed by centrifugation at 14,000× *g* for 20 min. This step was repeated 3 times. Then, 200 µL of 50 mM NH_4_HCO_3_ buffer was added and centrifuged at 14,000× *g* for 20 min, which was repeated twice. Subsequently, 4 µL of NH_4_HCO_3_ buffer containing trypsin (1:50) was added, and the mixture was incubated at 37 °C for 16 h. After transferring the mixture to a new collection tube and centrifuging at 14,000× *g* for 15 min, 40 μL of 50 mM NH_4_HCO_3_ buffer was added and centrifuged at 14,000× *g* for 30 min. The filtrate was collected in a new tube, and the sample was desalinated and freeze-dried. The resulting product was dissolved in 0.1% FA, and the peptide concentration was determined by measuring the absorbance at OD 280.

For high-performance liquid chromatography (HPLC), the HPLC system was used for chromatographic separation. Buffer A comprised 0. 1% formic acid, and buffer B solution comprised 0. 1% formic acid acetonitrile (84% acetonitrile). The chromatographic column was balanced with 95% buffer A. The sample was injected into the chromatographic column for gradient separation, and the flow rate was 300 nL/min. The liquid phase separation gradient was as follows: 0 min–2 min, with buffer B’s linear gradient ranging from 5% to 10%; 2 min–45 min, with buffer B’s linear gradient ranging from 10% to 30%; 45 min–55 min, with buffer B’s linear gradient ranging from 30% to 100%; and 55 min–60 min, with buffer B’s linear gradient maintained at 100%.

For high-resolution mass spectrometry PRM/MS analysis, the samples separated by HPLC were analyzed using PRM mass spectrometry with a Q-Exactive HF mass spectrometer (Thermo Scientific, Waltham, MA, USA) provided by AIMSMASS Co., Ltd. (Shanghai, China). Briefly, the analysis time: 60 min; and the detection mode: positive ion. The scanning range of the first stage mass spectrometry is 300–1800 *m*/*z*, and the resolution of the mass spectrometer is 60,000 (*m*/*z* 200). The AGC target is as follows: 3 × 10^6^, maximum IT: 200 ms. After each first-order MS scan (full MS scan), 20 PRM scans (MS2 scans) were collected according to the inclusion list. The isolation window is as follows: 1.6 Th; MS resolution: 30,000 (*m*/*z* 200); AGC target: 3 × 10^6^; maximum IT:120 ms; MS2 Activation Type: HCD; and normalized Collision energy: 27. All of the samples were tested using PRM, and the data contained in the original PRM file were finally analyzed finally using Skyline 22.2.0.

### 4.5. Primary BAECs Culture

Young Sprague Dawley (SD) rats, aged under 4 weeks, underwent perfusion using sterile 0.9% sodium chloride solution. The brain artery tissues were carefully separated and cut into pieces. The tissues were transferred into solutions containing type-II collagenase at a concentration of 2 mg/mL (dissolved in PBS with 1:1) and placed in a constant temperature bath at 37 °C. The tube containing the tissues was shaken every 5 min during a 30 min digestion and centrifugated at 1500 rpm for 10 min. Following the removal of the supernatant, the cells were gently resuspended using endothelial cells medium (ECM) (Sciencell, San Diego, CA, USA). The cells were inoculated in T25 cell culture flasks and cultured in a constant temperature cell incubator. After 24 h, the medium was replaced, and cells between passages 3 and 8 were utilized for the experiments.

### 4.6. Adenovirus Transfection

The packaging of adenovirus was completed by Sangon Biotech Co., Ltd. (Shanghai, China) and transfected with reference to the instructions. The sequencing results showed that the plasmids and the mutations were successfully constructed (See Supplementary Figure S1) Briefly, the primary BAECs of 3–8 passages were inoculated in advance with a density of 50% before transfection. The adenovirus expressing Flag-VEGFR2^WT^, Flag-VEGFR2^Y797F^ and Flag-VEGFR2^S799A^ (MOI: 1~2) were added to the half volume medium without penicillin and streptomycin. We added another half volume medium after 4 h and changed it to a new fresh medium after 6–8 h. The expression of GFP protein at 24 h, 48 h, 72 h, and 96 h were, respectively, observed under an inverted fluorescence microscope to evaluate the transfection efficiency.

### 4.7. Establishment of H/R Injury

The H/R model of the BAECs was established through experiments. Briefly, the cells were washed with phosphate-buffered saline (PBS), and then the medium was replaced with glucose-free DMEM. The BAECs were then exposed to a hypoxic environment comprising 1% O_2_, 95% N_2_, and 4% CO_2_ for 8 h. After exposure to hypoxia, the cells were switched to a normal ECM medium and cultured at 37 °C under normoxic conditions (37 °C, 95% O_2_ and 5% CO_2_) for 6 h. The BAECs in the control group were maintained under normoxic conditions without experiencing hypoxia.

### 4.8. Primary BAECs Proliferation Assay

The proliferation of BAECs was assessed using the cell counting kit (CCK-8). Following transfection with various plasmids for 72 h, BAECs were seeded into 96-well plates. Subsequently, the cells were subjected to hypoxic conditions (1% O_2_, 95% N_2_, and 4% CO_2_) and incubated in an incubator for 24 h with or without NaHS (50, 100, and 200 μmol/L). The absorbance at 450 nm was then measured using a microplate reader to evaluate the proliferation of each group.

### 4.9. Determination of the LDH LEVEL

The LDH levels in the BAEC culture supernatant were determined using a commercial assay kit. Briefly, the prepared BAEC suspension was centrifuged at 10,000 rpm for 10 min, and a microplate reader was used to detect the LDH levels at 450 nm, according to the protocol of the LDH assay kit.

### 4.10. Western Blot

Following the respective treatments, the cells were washed twice with ice-cold phosphate-buffered saline (PBS) (Servicebio, Wuhan, China) and then lysed in a cell lysis buffer. The protein concentration was determined using the BCA protein assay kit (Beyotime, Nanjing, China). Subsequently, a protein sample was combined with 5× sample buffer (4:1) and heated in boiling water for 10 min. The proteins were separated using 8% or 10% SDS-PAGE (Beyotime, Nanjing, China), transferred to polyvinylidene fluoride (PVDF) membranes (Millipore, Boston, MA, USA), and incubated with blocking buffer (0.05% Tween-20 PBS with 5% non-fat milk) for 2 h. Immunoblots were then exposed to the designated primary antibodies overnight at 4 °C, followed by the appropriate horseradish peroxidase-conjugated (HRP) secondary antibody (ZSGB-BIO, Beijing, China) for 2 h at room temperature. Immunodetection was visualized using enhanced chemiluminescence (Pierce, Edgewood, WA, USA). Autoradiographs were scanned using Tanon-5200 Image Quant LAS 4000 mini (Tanon Life Science, Shanghai, China). The density of the specific bands was quantified using Image J 1.54f (National Institutes of Health, New York, NY, USA).

### 4.11. Wound Healing Assay

The wound healing assay was used to detect the migration ability of cells. Briefly, BAECs were seeded evenly in 6-well plates at 70–90% confluency. Then, the cells were grouped and treated for the desired time. Then, the cells were treated with mitomycin (1 μg/mL) for 1 h in advance to inhibit cell division. After that, the cell monolayer was scraped in a straight line to create a “scratch” with a 200 μL pipette tip. The debris was removed, and the edge of the scratch was smoothed by washing the cells twice with 1 mL of PBS. The 2 mL serum-free ECM medium was added with or without different treatment (200 μmol/L of NaHS and 40 ng/mL of VEGF 164), and images were captured using a computer-based microscopic imaging system at 0 and 24 h, respectively. Cell motility was evaluated according to the following formula: cell motility ratio = (distance at 0 h − distance at 24 h)/distance at 0 h.

### 4.12. Transwell Assay

The migratory ability of cells was also detected through the use of a transwell assay. BAECs previously transfected with different plasmids by adenovirus for 72 h were grouped and treated according to the protocol of the H/R model. After trypsinization, they were washed with PBS to remove the influence of fetal bovine serum (FBS). Subsequently, the cell pellet was resuspended in serum-free ECM to a density of 1 × 10^6^ cells/mL. A total of 25,000–50,000 cells were seeded in the upper chamber of a transwell filter to assess their migratory capacity. Subsequently, the cells were incubated at 37 °C in a 5% CO_2_ environment for 24 h. Following incubation, the medium in the upper chamber was discarded, and the upper chamber was then carefully removed from the 24-well plates. The filter was fixed in 90% alcohol for 30 min and hexamethyl pararosaniline staining for 10 min. Images were captured of representative fields of each filter, and the number of cells was quantified.

### 4.13. Tube Formation Assay

To detect the tube formation of BAECs, a tube formation assay was utilized. Following the transfection of BAECs with different plasmids via adenovirus, the cells were grouped and subjected to the H/R model protocol. Subsequently, the cells were evenly seeded in 24-well plates at a density of 1 × 10^5^ cells/well, which had been pre-coated with Matrigel (BD Bioscience, San Jose, CA, USA). After a 6 h incubation, the cells were observed under a microscope and photographed. Finally, Image J 1.54f was utilized to quantify the cell branch points in the images.

### 4.14. Statistical Analysis

All of the data are presented as mean ± standard deviation (SD). One-way analysis of variance (one-way ANOVA) was used for multi-group comparisons. The results were considered statistically significant for *p*-value < 0.05.

## 5. Conclusions

In conclusion, our studies demonstrate for the first time that H_2_S exerts protective and pro-angiogenic effects by promoting the phosphorylation of VEGFR2 at Tyr797 and Ser799, with the latter being a novel phosphorylation site of VEGFR2. These findings suggest that H_2_S has a beneficial role in promoting angiogenesis through the activation of VEGFR2 and may provide the basis for the application of H_2_S in the therapy of ischemia stroke.

## Data Availability

The datasets used and/or analyzed during the current study are available from the corresponding author upon reasonable request.

## Supplementary Materials

The supporting information can be downloaded at: https://www.mdpi.com/article/10.3390/ijms25084340/s1.
