# Supplementary material for: Hydrogen Sulfide Exerted a Pro-Angiogenic Role by Promoting the Phosphorylation of VEGFR2 at Tyr797 and Ser799 Sites in Hypoxia–Reoxygenation Injury
Source: Int J Mol Sci. 2024 Apr 14;25(8):4340. doi: 10.3390/ijms25084340 (PMC11050214; doi:10.3390/ijms25084340)
Supplement: Supplementary file 1 [file ijms-25-04340-s001.zip › Supplementary Materials-Figure S1.pdf]

SUPPLEMENTARY

MATERIALS

Hydrogen Sulfide Exerted a Pro-Angiogenic Role by Promoting the Phosphorylation of VEGFR2 at Tyr797 and Ser799 Sites in Hypoxia–Reoxygenation Injury

Sen Zhang et al.

A

VEGFR2 ^{Y797}

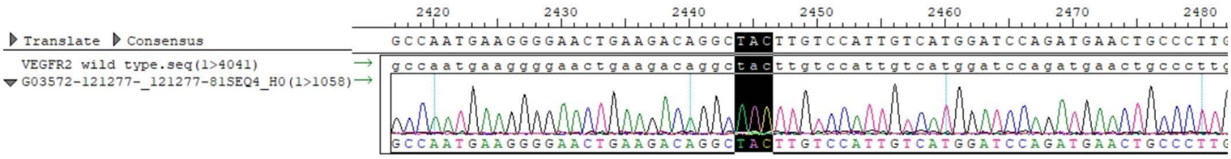

B

VEGFR2 ^{Y797F}

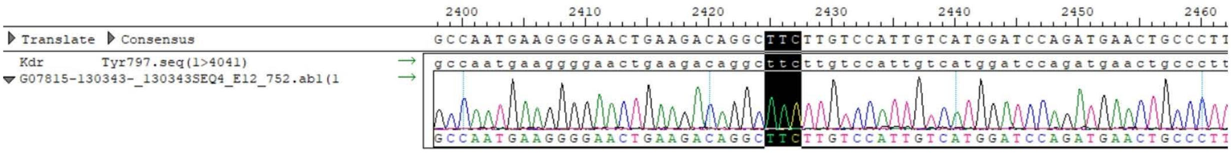

C

VEGFR2 ^{S799}

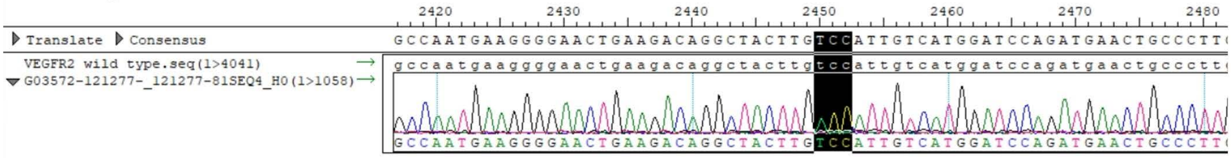

D

VEGFR2 ^{S799A}

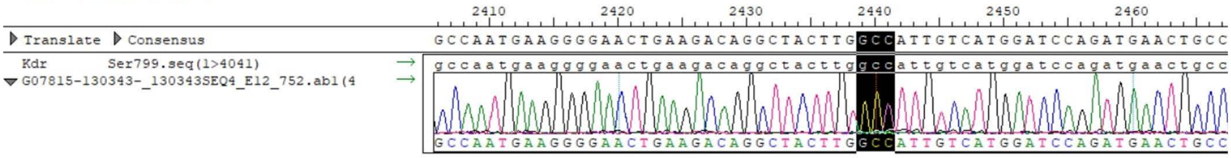

Figure S1: The plasmid sequencing results.

(A): The sequencing result of VEGFR2^{Y797}.

(B): The sequencing result of VEGFR2^{Y797F}.

(C): The sequencing result of VEGFR2^{S799}.

(D): The sequencing result of VEGFR2^{S799A}.
